# What do Cochrane systematic reviews say about telemedicine for healthcare?

**DOI:** 10.1590/1516-3180.0177240419

**Published:** 2019-07-15

**Authors:** Carolina Dutra Queiroz Flumignan, Aline Pereira da Rocha, Ana Carolina Pereira Nunes Pinto, Keilla Machado Martins Milby, Mayara Rodrigues Batista, Álvaro Nagib Atallah, Humberto Saconato

**Affiliations:** I MD, PhD. Postdoctoral Student, Evidence-Based Health Program, Universidade Federal de São Paulo (UNIFESP), and Volunteer Researcher, Cochrane Brazil, São Paulo (SP), Brazil.; II MSc. Pharmacist and Doctoral Student, Evidence-Based Health Program, Universidade Federal de São Paulo (UNIFESP), and Volunteer Researcher, Cochrane Brazil, São Paulo (SP), Brazil.; III MSc. Physiotherapist and Doctoral Student, Evidence-Based Health Program, Universidade Federal de São Paulo (UNIFESP), São Paulo (SP); Professor, Department of Biological and Health Sciences, Universidade Federal do Amapá, Macapá (AP); and Volunteer Researcher, Cochrane Brazil, São Paulo (SP), Brazil.; IV BSc. Nurse and Doctoral Student, Evidence-Based Health Program, Universidade Federal de São Paulo (UNIFESP), and Volunteer Researcher, Cochrane Brazil, São Paulo (SP), Brazil.; V BSc. Librarian and Doctoral Student, Evidence-Based Health Program, Universidade Federal de São Paulo (UNIFESP), and Volunteer Researcher, Cochrane Brazil, São Paulo (SP), Brazil.; VI MD, MSc, PhD. Nephrologist and Full Professor, Discipline of Emergency and Evidence-Based Medicine, Escola Paulista de Medicina (EPM), Universidade Federal de São Paulo (UNIFESP), and Director, Cochrane Brazil, São Paulo (SP), Brazil.; VII MD, PhD. Adjunct Professor, Discipline of Emergency and Evidence-Based Medicine, Universidade Federal de São Paulo (UNIFESP), and Researcher, Cochrane Brazil, São Paulo (SP), Brazil.

**Keywords:** Telemedicine, Electronic health records, Telerehabilitation, Telephone, Delivery of health care

## Abstract

**BACKGROUND:**

Telemedicine has emerged as a tool for overcoming the challenges of healthcare systems and is likely to become increasingly viable, since information and communication technologies have become more sophisticated and user-friendly.

**OBJECTIVE:**

We aimed to identify all Cochrane systematic reviews (CSRs) on telemedicine within healthcare and to summarize the current evidence regarding its use.

**DESIGN AND SETTING:**

Review of CSRs, developed at the Discipline of Emergency and Evidence-Based Medicine, Escola Paulista de Medicina, Universidade Federal de São Paulo.

**METHODS:**

We searched for studies that compared use of telemedicine with conventional treatment or management of diseases within healthcare. Diagnostic telemedicine studies or studies using automatic text, voice-text or even self-managed care were excluded. The main characteristics and the certainty of evidence were synthetized and critically discussed by all authors.

**RESULTS:**

We included 10 CSRs that investigated a broad range of diseases. There is still insufficient evidence to determine what types of telemedicine interventions are effective, for which patients and in which settings, and whether such interventions can be used as a replacement for the standard treatment. Harm relating to telemedicine technologies needs to be better investigated and addressed.

**CONCLUSION:**

Telemedicine might be an excellent way to facilitate access to treatment, monitoring and dissemination of important clinical knowledge. However, given the recognition of systematic reviews as the best evidence resource available for decision-making, further randomized controlled trials with stricter methods are necessary to reduce the uncertainties in evidence-based use of telemedicine.

## INTRODUCTION

Telemedicine is the use of information and communication technologies to provide healthcare services, especially when distance is a critical factor in accessing the healthcare provider.^1^ Since 1879, the year in which the first medical telephone consultation was documented in an article published in the Lancet journal,^2^ telemedicine and its use as a tool for improving healthcare have been continually expanding.

Although telemedicine is not a novel activity, it has over the past few years emerged as an alternative tool for addressing the challenges of universal healthcare systems, such as expansion of access to specialized healthcare services in regions that lack these resources.^3^ This type of technology not only has the direct impact of improving access to treatment but also positively affects the environment while decreasing emissions of pollutants through reduction of staff and patients’ need to travel. This is favored through remote care,^4^ given that the physical distance between the patient and the provider of the intervention always exists.^5^

Telemedicine provides a virtual environment that enables remote interaction between healthcare professionals and their patients, and among healthcare professionals themselves. This particular characteristic, which goes beyond conventional standards, changes paradigms and has ethical and legal implications in each country where it is used, especially in relation to the confidentiality of patients’ data.^6^ The role of telemedicine within healthcare professionals’ continuing education, research and evaluation is emphasized. This not only improves healthcare access for patients but also enables access to high-quality information for healthcare professionals located in remote settings.^7^

In the United States, it has been estimated that more than a quarter of consultations are undertaken through telephone calls.^8^ Telemedicine has been evolving and progressing through the advent of mobile phone applications such as Doctor On Demand, HealthTap and Pingmd.

The territorial extent of Brazil is huge, with thousands of isolated, difficult-to-access places and unequal distribution of good-quality medical resources. These characteristics put the right to universal, comprehensive and equitable healthcare services at risk and indicate that there is great potential for expansion of telemedicine in this country.^1^ On the other hand, the benefits and harm relating to its use have not yet been established.

## OBJECTIVE

The aim of this study was to identify all Cochrane systematic reviews (CSRs) on telemedicine within healthcare and to summarize the current evidence regarding its use.

## METHODS

### Design

Review of Cochrane systematic reviews (SRs).

### Setting

Discipline of Urgency and Evidence-Based Medicine, Escola Paulista de Medicina (EPM), Universidade Federal de São Paulo (UNIFESP), Brazil.

### Criteria for including reviews

#### Types of studies

We considered the latest versions of the published Cochrane SRs. We did not include any protocols or any SRs that had been withdrawn from the Cochrane Database of Systematic Reviews.

#### Types of participants

We considered all patients who made use of any category of telehealth intervention, without any restriction relating to type of intervention or the age or sex of the participants.

#### Types of interventions

We considered any category of telemedicine intervention for treatment or management of diseases within healthcare. We excluded studies that assessed the effect of telemedicine for diagnostic purposes or studies that used automatic text or voice-text or even self-managed care.

#### Types of outcomes

We considered any outcomes that had been assessed and reported by the authors of the SRs included.

### Search strategy

We conducted an unrestricted systematic search within the Cochrane Database of Systematic Reviews (via Wiley) on February 26, 2019. The search strategy is presented in Table 1.

**Table 1.** Search Strategy in Cochrane Library

Cochrane Library (26/02/2019)
#1 Mesh: [Telemedicine] = 1,982
#2 (Mobile Health) OR (Health, Mobile) OR mHealth OR Telehealth OR eHealth = 4,596
#3 #1 OR #2 = 5,946
#4 #3 in Cochrane Reviews = 326

### Selection of systematic reviews

The selection phase consisted of reading of all the abstracts retrieved, by three researchers independently (APR, ACPNP, KMM), to check their eligibility in relation to the inclusion criteria. Any disagreement was resolved through reaching a consensus among these three authors or by consulting a fourth author (CDQF).

## RESULTS

Out of the 326 reviews that had been included in the primary analysis, we selected 10 SRs (published between 2011 and 2016) containing data on 53 randomized controlled trials (RCTs) with 6,836 participants, which had assessed the effectiveness and safety of telemedicine within healthcare. These SRs addressed the effects of telemedicine regarding heart-failure patients (n = 1),^7^ chronic obstructive pulmonary disease (n = 1),^9^ treatment and management of asthma (n = 2),^10^^,^^11^ low vision (n = 1),^12^ multiple sclerosis (n = 1),^13^ stroke (n = 1),^14^ parents of high-risk newborns (n = 1),^15^ HIV patients (n = 1),^16^ and children and adolescents with chronic pain (n = 1).^18^ The main results are shown in Table 2.

**Table 2.** Characteristics of studies included

Intervention	Comparison	Population	Benefits and harms	Certainty of evidence
Telehealthcare with input from a professional^9^	Standard care	Patients of any age, gender, ethnicity or language with COPD diagnosed by a clinician (n = 1,004)	- Total number of exacerbations recorded showed borderline statistical significance - Significantly fewer episodes of exacerbation per month. - Number of days free from exacerbations after one year was higher within the intervention group (30%) - There was a minimally clinically significant change regarding quality of life. - Fewer visits to the emergency service - Lower hospital admission rate - No difference in mortality rate between the groups.	- not assessed
Remote check-ups for asthma^10^	Standard check-up	Adults or children with asthma (n = 2,100)	- Greater need for oral corticosteroid intake in comparison with the control group - Fewer exacerbation than in face-to-face check-up group. - No difference in score relating to the Asthma Control Questionnaire - Lung function improvement (reported in one study) - Higher number of serious adverse events reported. Exacerbation requiring hospital admission was the most frequent of the events - Quality of life score similar to that of the control group (Asthma Quality of Life Questionnaire)	- low level - low level - moderate level - moderate level - not assessed - not assessed
Home telemonitoring and remote feedback between clinic visits for asthma^11^	Standard care	Adults or children with a diagnosis of asthma (n = 2,268)	- Number of episodes of exacerbation requiring oral corticosteroids was similar to that of standard care - No difference between telemonitoring and usual monitoring, regarding exacerbations requiring hospital admission - Improvement in the asthma quality of life score - Improvement of lung function - Telemonitoring did not lead to any clear increase or decrease in the number of unscheduled healthcare visits	- low level - moderate level - low level - moderate level - very low level
Telerehabilitation^12^	Standard rehabilitation	People with low vision or visual function loss due to any ocular condition	- Not assessed (no studies included)	- not assessed
Telerehabilitation^13^	Standard rehabilitation	Patients diagnosed with multiple sclerosis (> 18 years old) (n = 531)	- Reduction of short-term disability and symptoms such as fatigue - Long-term improvement in functional activities and impairments (such as fatigue, pain and insomnia) - Social re-integration measured through quality of life and psychological outcomes. - No adverse events relating to telerehabilitation were reported	- low level
Telerehabilitation^14^	Standard care	Patients diagnosed with stroke (n = 933)	- No difference in independence regarding activities of daily living and upper-limb function. - Insufficient data to draw conclusions regarding the effects of the intervention on mobility, health-related quality of life or participant satisfaction. - No adverse events relating to telerehabilitation were reported.	- not assessed
Baby Carelink^15^	Standard care	Parents of high-risk newborns in NICU (n = 56)	- No difference between groups regarding the length of hospital stay	- very low
Interventions delivered by telephone^16^	Standard care	HIV-infected patients (n = 1,381)	- No difference in adherence to antiretroviral medication - No difference in depressive symptoms	- low quality - low quality
Non-invasive telemonitoring^17^	Standard care	Patients with heart failure (n = 3,860)	- Reduction in all-cause mortality rates - Reduction in heart failure-related hospitalizations - No difference in reduction of risk of all-cause hospitalizations	- moderate level - moderate level - very low level
Structured telephone^17^	Standard care	Patients with heart failure (n = 9,332)	- Reduction in all-cause mortality rates - Reduction in heart failure-related hospitalizations - No difference in reduction of risk of all-cause hospitalizations	- moderate level - moderate level - very low level
Psychological therapies delivered remotely^18^	Face-to-face psychological therapy or waiting list	Children and adolescents (0 to 18 years old) with chronic pain (n = 371)	- Severity of headache pain reduced post-treatment - Pain intensity reduced post-treatment in mixed pain conditions (i.e. recurrent abdominal pain or musculoskeletal pain) - At follow-up: no difference in headache conditions - No difference in depression in headache group	- not assessed

NICU = neonatal intensive care unit; COPD = chronic obstructive pulmonary disease; n = number of participants.

### Home telemonitoring and remote feedback for asthma

This review^11^ included 18 parallel RCTs with a total of 2,268 participants. The results from this review showed that there was no difference between home telemonitoring and the usual monitoring relating to exacerbations that would require use of oral corticosteroids (odds ratio [OR] 0.93; 95% confidence interval [CI] 0.60 to 1.44; 466 participants; four studies; I^2^ = 0%; low quality of evidence). In relation to exacerbations requiring hospitalization, there was uncertainty regarding the benefit or harm, compared with the standard monitoring procedure (OR 0.56; 95% CI 0.21 to 1.49; 1,042 participants; 10 studies; I^2^ = 45%; moderate quality of evidence).

Overall, the evidence relating to asthma control was very weak due to the inconsistency of outcomes. The patients in the telemedicine groups scored better in the Asthma Quality of Life Questionnaire than did those who were monitored using standard protocols (median difference [MD] 0.23; 95% CI 0.01 to 0.45; 796 participants; six studies; I^2^ = 54%; low quality of evidence). Adverse events, whether serious or non-serious, were not reported in any of the studies included in this review. Small benefits regarding quality of life were observed, although the studies were unblinded. Some benefits regarding lung function were reported, but the effects were uncertain due to possible attrition bias.

The quality of evidence was downgraded by the SR authors because of imprecision, inconsistency, publication bias and the risk of bias. The authors of the review^11^ concluded that the current evidence did not support widespread implementation of telemonitoring, with feedback between visits to asthma clinics.

For further details, the full content of this review can be accessed through: https://www.cochranelibrary.com/cdsr/doi/10.1002/14651858.CD011714.pub2/full.

### Remote check-ups for asthma

Six RCTs with 2,100 participants were included in this review.^10^ These RCTs compared remote check-ups using various forms of telehealth technology (telephone calls or video-conferencing) versus standard face-to-face check-ups. The results from a cluster study and an oral steroid tapering study were also included, but they were reported separately.

The effects from use of telehealth among people who required oral corticosteroids to treat exacerbations were greater in the telehealth group than in the control group, although the confidence intervals were very wide due to the small number of events (OR 1.74; 95% CI 0.41 to 7.44; 278 participants; low quality of evidence). Moreover, the cluster study showed that there were positive effects in the face-to-face check-up groups (OR 1.43, 95% CI 1.04 to 1.97).

The effect of exacerbation events that needed emergency department visits favored the face-to-face check-up groups, but this was uncertain due to the small number of events (OR 2.60; 95% CI 0.63 to 10.64; 651 participants; three studies; low quality of evidence). However, neither the RCTs (Peto OR 0.63; 95% CI 0.06 to 6.32; 651 participants; three studies; low quality of evidence) nor the cluster implementation study (Peto OR 2.18; 95% CI 0.83 to 5.69; 1,213 participants; one study) showed any statistically significant benefits, compared with face-to-face check-ups, regarding exacerbations that required hospital admission.

The authors of this review reported that there were no differences relating to the scores obtained in the Asthma Control Questionnaire between the remote and face-to-face groups (MD 0.07; 95% CI −0.35 to 0.21; 146 participants; one study; moderate quality of evidence). There were no differences between the two groups regarding asthma-related quality of life, as determined through the Asthma Quality of Life Questionnaire (MD 0.08; 95% CI −0.14 to 0.30; 544 participants; three studies; moderate quality of evidence).

The results relating to unscheduled healthcare visits were imprecise with few events and, therefore, it was impossible to draw a conclusion. Lung function assessed by means of forced expiratory volume in the first second (FEV1) was only reported in one study. The authors of this review^10^ identified enhancement of lung function in the remote check-up group, compared with the face-to-face group (MD 166.76; 95% CI 78.03 to 255.50; 253 participants; one study; moderate quality of evidence). No adverse events, whether serious or non-serious, were recorded in any of the studies included in this review.

All the available evidence was based on small RCTs. Outcomes relating to lung function, asthma control and exacerbations requiring oral corticosteroids were only reported in one study. The evidence was downgraded by the SR authors to moderate quality regarding asthma-related quality of life and asthma control because of the lack of blinding of the participants and outcome assessments. The evidence regarding the lung function was downgraded because of imprecision relating to the small sample size. The data on the other outcomes were of low quality due to the wide CIs, small number of events and risk of bias.

For further details, the full content of this review can be accessed through: https://www.cochranelibrary.com/cdsr/doi/10.1002/14651858.CD011715.pub2/full.

### Telehealthcare for chronic obstructive pulmonary disease (COPD)

This review^9^ included 10 RCTs comparing telehealth with the usual care among 1,004 patients who had been diagnosed with COPD.

Total number of exacerbations was only analyzed in one RCT, with borderline statistical significance (P = 0.06). In another trial, it was reported that the mean number of exacerbations per month was significantly higher among the controls than in the telehealthcare group (0.78 ± 0.77 and 0.23 ± 0.38, respectively; P < 0.0001). The number of days that were free from exacerbation after one year was higher in the intervention group (30%) than in the control group (5%).

Quality of life was assessed through scores from the validated St George Respiratory Questionnaire (SGRQ), and improvement of quality of life showed slight clinical significance with very wide confidence intervals (MD -6.57; 95% CI -13.62 to 0.48; 253 participants; two studies). The patients who received telehealthcare were much less likely to attend the emergency department than were the individuals in the control group (OR 0.27; 95% CI 0.11 to 0.66; 449 patients; three studies), and the number of patients with one or more hospital admissions was lower in the telehealthcare group (OR 0.46; 95% CI 0.33 to 0.65; P < 0.00001; 604 patients; four studies). There was no difference in mortality rate between the groups (OR 1.05; 95% CI 0.63 to 1.75; P = 0.86; 503 patients; three studies). There were no differences between the groups regarding lung function or patient satisfaction. The authors of this review encouraged support for telehealthcare, even though the evidence came from very heterogeneous studies.

For further details, the full content of this review can be accessed through: https://www.cochranelibrary.com/cdsr/doi/10.1002/14651858.CD007718.pub2/full.

### Telerehabilitation for people with low vision

This review^12^ did not find any RCTs that had evaluated the effectiveness and/or safety of telerehabilitation for people with low vision. However, given the growing interest in telemedicine and the burden of low vision, it was recommended that pilot studies should be conducted in the future in order to explore the potential for telemedicine to provide rehabilitation for people with low vision.

For further details, the full content of this review can be accessed through: https://www.cochranelibrary.com/cdsr/doi/10.1002/14651858.CD011019.pub2/full?highlightAbstract^­^=telerehabilit%7Cwithdrawn%7Ctelerehabilitation.

### Telerehabilitation for patients with multiple sclerosis

This review^13^ included nine RCTs (n = 531 participants) that assessed a wide variety of telerehabilitation methods among adults with multiple sclerosis. The patients’ ages ranged from 41 to 52 years (mean of 46.5 years). The mean number of years since the patients received their diagnosis ranged from 7.7 to 19 years (mean of 12.3 years).

The telerehabilitation interventions included physical activity and educational, behavioral and symptom management programs. The duration of the interventions ranged from one to six months (median of 12 weeks). The main outcomes evaluated were the following: functional activities; improvement in symptoms or impairments (pain, fatigue, spasms frequency, spasticity and others); quality of life; and psychosocial outcomes.

No quantitative analysis could be conducted in this review,^13^ due to clinical and methodological heterogeneity. Overall, the review found that there was a low level of certainty regarding telerehabilitation interventions for reducing short-term disability and symptoms, such as fatigue in patients with multiple sclerosis. In longer-term follow-ups, there was also a low level of certainty regarding telerehabilitation for improving functional activities and impairments (such as fatigue, pain and insomnia); and regarding participation, as measured in terms of quality of life and psychological outcomes. Regarding safety, the studies included did not report any adverse event relating to telerehabilitation.

Multiple sclerosis is a complex condition and the range of telerehabilitation interventions and their prescription requirements can vary from person to person and are difficult to standardize. Factors such as the patients’ functional abilities, personal characteristics and comorbidities and the characteristics of the healthcare system may influence patients’ outcomes. The interaction of these factors with rehabilitation strategies and their impact on patients’ outcomes is still little understood. However, because the multiple sclerosis population is young and has high rates of internet use, these patients are likely to be receptive to telerehabilitation.

In addition to the limited number of studies in this review and the high heterogeneity among these studies, methodological weaknesses were identified in them (underpowered data due to small sample sizes, high risk of bias, short follow-up periods, lack of rigorous methodology and differences in outcome measurements).

For further details, the full content of this review can be accessed through: https://www.cochranelibrary.com/cdsr/doi/10.1002/14651858.CD010508.pub2/full?highlightAbstract­=telerehabilit%7Cwithdrawn%7Ctelerehabilitation.

### Telerehabilitation for stroke

Ten RCTs (n = 933) were included in this review.^14^ Regarding age, the patients were in their 50s to 70s. All interventions were delivered in the patients’ own homes. The patients were generally in the chronic phase following a stroke.

The telerehabilitation interventions included use of telephones, videoconferencing hardware and software, desktop videophones, in-home messaging devices, video recordings, emails, online chat programs and online resource rooms. The intervention approaches included upper limb training, lower limb and mobility retraining, case management and caregiver support. Several outcomes were evaluated, such as physical function, independence in activities of daily living, quality of life and participant satisfaction.

Pooled data from 661 participants did not show any statistically significant results regarding independence in activities of daily living when a case management intervention was evaluated (standardized mean difference [SMD] 0.00; 95% CI -0.15 to 0.15). No statistically significant results regarding upper limb function (based on two studies with 46 participants: MD 3.65; 95% CI -0.26 to 7.57) were observed when computer software was used to remotely retrain upper limb function.

Insufficient evidence was found to draw any conclusions regarding the effects of the intervention on mobility, health-related quality of life or participant satisfaction with the intervention. No adverse events were reported within the studies.

Overall, telerehabilitation offers great potential as an intervention to be used in addition to current therapies or as a therapy for patients with difficulty in accessing places where face-to-face rehabilitation can be provided. However, it is still important to investigate whether there are any differences in the same therapy between delivery face-to-face and delivery via telecommunication.

For further details, the full content of this review can be accessed through: https://www.cochranelibrary.com/cdsr/doi/10.1002/14651858.CD010255.pub2/full?highlightAbstract­=telerehabilit%7Cwithdrawn%7Ctelerehabilitation.

### Telemedicine for supporting parents or caregivers of high-risk newborns while hospitalized in an intensive care unit

The aim of this review^15^ was to assess the effects of use of telemedicine (Baby Carelink) to support the families of newborns, regarding the newborn’s length of hospital stay and the family’s satisfaction while the newborn was hospitalized in an neonatal intensive care unit (NICU), compared with those that received standard care without access to this telemedicine program. The study included one RCT (n = 56 newborn infants). The Baby Carelink program consisted of use of multimedia and videoconference devices that provided the families with the infant’s daily clinical progress and also enabled provision of clinical information, communication through a message center, release preparation, viewing of the infant and a family room.

The results from the review showed that the lengths of hospital stay were similar in the telemedicine group (68.5 days; standard deviation [SD] 28.3 days) and the control group (70.6 days; SD 35.6 days; MD -2.10 days; 95% CI -18.85 to 14.65 days). The quality of the evidence was very low due to the small sample size and imprecision of the effect estimates.

The participants formed a very specific group: families living within the urban area, with good internet access, who were competent in English and had higher economic status than other possible candidates. There was a withdrawal rate of 20% within the control group, since these newborns were transferred back to level-2 nurseries. The data regarding family satisfaction and other outcomes were insufficient for conducting proper analysis.

Thus, so far, there is not enough evidence to promote use of telemedicine to support parents or caregivers of newborns receiving intensive care as an effective procedure. However, this study dates back to the year 2000 and many technological resources have been developed since then. For this reason, we cannot rule out the idea that application of telemedicine to this kind of population now could have a different outcome.

For further details, the full content of this review can be accessed through: https://www.cochranelibrary.com/cdsr/doi/10.1002/14651858.CD006818.pub2/epdf/full.

### Telephone-delivered interventions for HIV-infected patients:

The aim of this review^16^ was to assess the effectiveness of interventions delivered by telephone, compared with standard care for HIV-infected patients. It included 11 RCTs, with 1,381 participants.

The main findings were that there were no differences between the intervention and control groups regarding adherence to antiretroviral medication, with low quality of evidence (3 studies; 191 participants; SMD 0.49; 95% CI -1.12 to 2.11; P = 0.55), or regarding depressive symptoms, also with low quality of evidence (3 studies; 447 participants; SMD 0.02; 95% CI -0.18 to 0.21; P = 0.85). In relation to all other information (reduction of risky sexual behavior, virological outcomes and psychiatric symptoms other than depression), there was insufficient data to provide meta-analyses.

For further details, the full content of this review can be accessed through: https://www.cochranelibrary.com/cdsr/doi/10.1002/14651858.CD009189.pub2/full.

### Structured telephone support or non-invasive telemonitoring for patients with heart failure

This review^17^ addressed how telemonitoring and structured telephone support could help patients with heart failure in relation to undesirable outcomes such as hospitalization and death. The difference between these two interventions lay in the manner in which they were used: structured telephone support only used the technology of transmission by telephone to collect patient data; while telemonitoring involved multiple ways of transmission, such as Bluetooth, satellite and wireless technologies. For this review, 25 studies evaluated structured telephone support interventions (9,332 participants) and 18 studies assessed non-invasive home telemonitoring (3,860 participants).

The main findings from this review regarding non-invasive telemonitoring were that it gave rise to reductions in the all-cause mortality rates (17 studies; 3,740 participants; relative risk [RR] 0.80; 95% CI 0.68 to 0.94; P = 0.0057); and reductions in heart failure-related hospitalizations (8 studies; 2148 participants; RR 0.71; 95% CI 0.60 to 0.83; P = 0.000013). Structured telephone support also reduced all-cause mortality (22 studies; 9,222 participants; RR 0.87; 95% CI 0.77 to 0.98; P = 0.017) and had a positive impact regarding reduction of hospitalizations caused by heart failure (16 studies; 7,030 participants; RR 0.85; 95% CI 0.77 to 0.93; P = 0.00047). All of these outcomes were graded as presenting moderate quality of evidence.

This review did not show any difference regarding reduction of the risk of all-cause hospitalizations, (structured telephone support: 16 studies; 7,216 participants; RR 0.95; 95% CI 0.90 to 1.00; P = 0.055; and non-invasive telemonitoring: 13 studies; 3,332 participants; RR 0.95; 95% CI 0.89 to 1.01; P = 0.033), with very low quality of evidence. The outcomes of length of stay, quality of life related to the health condition, cost effectiveness and treatment adherence were not described consistently, which hindered development of a meta-analysis. The funnel plot analyses demonstrated that there was strong evidence of publication bias.

For further details, the full content of this review can be accessed through: https://www.cochranelibrary.com/cdsr/doi/10.1002/14651858.CD007228.pub3/full.

### Psychological therapies (remotely delivered) for management of chronic and recurrent pain in children and adolescents

This review^18^ examined the effectiveness of treatments performed remotely (via the internet, telephone and audiotapes, among other methods), in comparison with face-to-face psychological therapy or waiting-list control in a population from 0 to 18 years of age with chronic and recurrent pain. It included 8 studies, with 371 participants.

The severity of headache pain was reduced through remote psychological treatments (6 studies; 247 participants; RR 2.65; 95% CI 1.56 to 4.50; P = 0.00030; number needed to treat to benefit [NNTB] 2.88). For mixed pain conditions, i.e. musculoskeletal pain or abdominal pain with recurrence, there was a beneficial effect regarding reduction of pain intensity post-treatment (3 studies; 131 participants; standardized mean difference [SMD] -0.61; 95% CI -0.96 to -0.25; P = 0.00074).

At follow-up, however, no statistical difference in headache conditions was achieved (3 studies; 85 participants; RR 1.56; 95% CI 0.67 to 3.68; P = 0.30). This was also observed regarding the outcome of depression, when analyzed in the same headache groups (2 studies; 103 participants; SMD 0.02; 95% CI -0.38 to 0.43; P = 0.91). For headache and mixed conditions, there were no beneficial effects from the therapies included as interventions in this review (2 studies; 94 participants; SMD -0.50; 95% CI -1.02 to 0.02; P = 0.06). No data were available in relation to any other outcomes, or in relation to adverse events.

The authors of this review did not use GRADE (the grading of recommendations, assessment, development and evaluations recommended by Cochrane) in the assessment of risk of bias because of the lack of information in the studies included. However, they classified most of the studies as presenting “low risk” or “unclear risk” of bias.

For further details, the full content of this review can be accessed through: https://www.cochranelibrary.com/cdsr/doi/10.1002/14651858.CD011118.pub2/full.

## DISCUSSION

The use of telemedicine is likely to become increasingly viable as information and communication technologies within healthcare become more sophisticated and user friendly. The driving force behind this is the need for an alternative to face-to-face interventions that enables service delivery in the natural environment, i.e. in patients’ homes.

For pulmonary care, telemedicine has become an important alternative to the standard care. Chronic obstructive pulmonary disease (COPD) is the third leading cause of death worldwide.^19^^,^^20^ Moreover, it has been estimated that around 300 million people around the world are affected by asthma. Thus, there is a need to explore the available evidence regarding the benefits or harm from telemedicine care. Telemedicine seems to reduce hospitalization and emergency department visits, and it possibly has an impact on the quality of life of patients with COPD.^21^^,^^9^

Telerehabilitation is an emerging method that extends rehabilitative care beyond the hospital, using telecommunication technology at home or in the community.^22^ Overall, a wide range of telerehabilitation methods have been studied, but the evidence for their effectiveness remains unclear. In this review, we found that the telerehabilitation interventions evaluated were complex, with various rehabilitation components that included physical activity and educational, behavioral and symptom management programs. These interventions had different purposes and used different technologies, and therefore no single definitive overall conclusion was possible.

We also performed a broad search in the Medline database via PubMed on March 12, 2019, in order to find other reviews. The PubMed search strategy is provided in Appendix A. Our search retrieved 1,274 reviews. We looked for SRs on asthma, chronic obstructive pulmonary disease, stroke, low vision, multiple sclerosis, parents of high-risk newborns, HIV patients, heart-failure patients or children and adolescents with chronic pain, in order to make comparisons with our Cochrane database findings.

In general, the Cochrane reviews had broader searches and identified greater numbers of randomized trials than were described in other reviews^23^^,^^24^ published within the same period as the Cochrane SRs (2011-2016). However, it seems that the Cochrane and non-Cochrane reviews came to similar conclusions: namely, that the evidence is currently insufficient to draw any conclusions regarding the effectiveness of telemedicine.^25^ Nonetheless, a recent review (found in Medline) that was published after 2016 showed that telemedicine was a promising alternative tool for improving motor function in patients who had suffered a stroke.^26^

Furthermore, although the purpose of telemedicine is to reduce costs and overcome some barriers such as availability of transportation, hospital costs and ability to make visits to healthcare professionals, no Cochrane reviews have identified any trials on its cost-effectiveness. Establishment of telemedicine services can be expensive due to the costs of equipment, training and ongoing technical support. Therefore, it is important to determine whether, once telemedicine services have been established, they should be used as an alternative or as a supplement to the conventional therapy that is delivered face-to-face.

It is also relevant to note that the use of technology to facilitate communication may, on the other hand, lead to miscommunication. For example, healthcare professionals may make errors relating to their assessments of patients, or these patients may misunderstand the advice or instructions provided by the healthcare professional. Therefore, not only the benefits, but also the harm associated with telemedicine needs to be addressed.

Overall, there is still insufficient evidence regarding what types of telemedicine interventions are effective, and for which patients in which setting. Here, we can also highlight the lack of robust, methodologically strong studies evaluating the effectiveness of the different technologies relating to telemedicine interventions. Researchers should ensure that trials are adequately powered, developed with high methodological quality and reported in compliance with the Consolidated Standards of Reporting Trials (CONSORT) guidelines.

## CONCLUSION

In the data universe of the Cochrane Library, we found 10 Cochrane systematic reviews relating to the use of telemedicine within healthcare. The quality of the evidence reported was too low to support or refute the use of this technology within clinical practice as an effective intervention for replacement of standard treatment. The best evidence available was of moderate quality, relating to the effectiveness of telemedicine among patients with heart failure, for reducing the risks of heart failure-related hospitalization and all-cause mortality.

Given the growing interest in telemedicine and the recognition of the Cochrane Library as the best evidence resource available for decision-making, further RCTs with stricter methods are necessary in order to address many structured clinical questions and inform better systematic reviews. Such RCTs will be able to evaluate the efficacy, effectiveness, efficiency and safety of telemedicine. In this manner, the uncertainties regarding the therapeutic, managerial, ethical and economic aspects of evidence-based telemedicine could be reduced. Moreover, telemedicine may be an excellent way to facilitate access to treatment and monitoring and to disseminate important clinical knowledge among healthcare professionals.

## Appendix A. MEDLINE via PubMed search strategy

(((“Telemedicine”[Mesh]) OR (mobile health) OR (health, mobile) OR telehealth OR ehealth))) AND ((((systematic review[ti] OR systematic literature review[ti] OR systematic scoping review[ti] OR systematic narrative review[ti] OR systematic qualitative review[ti] OR systematic evidence review[ti] OR systematic quantitative review[ti] OR systematic meta-review[ti] OR systematic critical review[ti] OR systematic mixed studies review[ti] OR systematic mapping review[ti] OR systematic cochrane review[ti] OR systematic search and review[ti] OR systematic integrative review[ti]) NOT comment[pt] NOT (protocol[ti] OR protocols[ti])) NOT MEDLINE[subset]) OR (Cochrane Database Syst Rev[ta] AND review[pt]) OR systematic review[pt])
